# Paternal Urinary Concentrations of Parabens and Other Phenols in Relation to Reproductive Outcomes among Couples from a Fertility Clinic

**DOI:** 10.1289/ehp.1408605

**Published:** 2015-03-13

**Authors:** Laura E. Dodge, Paige L. Williams, Michelle A. Williams, Stacey A. Missmer, Thomas L. Toth, Antonia M. Calafat, Russ Hauser

**Affiliations:** 1 Department of Epidemiology, and; 2 Department of Biostatistics, Harvard T.H. Chan School of Public Health, Boston, Massachusetts, USA; 3 Department of Obstetrics, Gynecology and Reproductive Biology, Harvard Medical School, Boston, Massachusetts, USA; 4 Channing Division of Network Medicine, Department of Medicine, Brigham and Women’s Hospital and Harvard Medical School, Boston, Massachusetts, USA; 5 Department of Obstetrics and Gynecology, Division of Reproductive Endocrinology and Infertility, Massachusetts General Hospital, Boston, Massachusetts, USA; 6 National Center for Environmental Health, Centers for Disease Control and Prevention, Atlanta, Georgia, USA; 7 Department of Environmental Health, Harvard T.H. Chan School of Public Health, Boston, Massachusetts, USA

## Abstract

**Background:**

Human exposure to phenols, including bisphenol A and parabens, is widespread. Evidence suggests that paternal exposure to environmental chemicals may adversely affect reproductive outcomes.

**Objectives:**

We evaluated associations of paternal phenol urinary concentrations with fertilization rate, embryo quality, implantation, and live birth.

**Methods:**

Male–female couples who underwent *in vitro* fertilization (IVF) and/or intrauterine insemination (IUI) cycles in a prospective study of environmental determinants of fertility and pregnancy outcomes were included. The geometric mean of males’ specific gravity–adjusted urinary phenol concentrations measured before females’ cycle was quantified. Associations between male urinary phenol concentrations and fertilization rate, embryo quality, implantation, and live birth were investigated using generalized linear mixed models to account for multiple cycles per couple.

**Results:**

Couples (*n* = 218) underwent 195 IUI and 211 IVF cycles. Paternal phenol concentrations were not associated with fertilization or live birth following IVF. In adjusted models, compared with the lowest quartile of methyl paraben, paternal concentrations in the second quartile were associated with decreased odds of live birth following IUI (adjusted odds ratio = 0.19; 95% CI: 0.04, 0.82).

**Conclusions:**

To our knowledge, these are some of the first data on the association of paternal urinary phenol concentrations with reproduction and pregnancy outcomes. Although these results do not preclude possible adverse effects of paternal paraben exposures on such outcomes, given the modest sample size, further understanding could result from confirmation using a larger and more diverse population.

**Citation:**

Dodge LE, Williams PL, Williams MA, Missmer SA, Toth TL, Calafat AM, Hauser R. 2015. Paternal urinary concentrations of parabens and other phenols in relation to reproductive outcomes among couples from a fertility clinic. Environ Health Perspect 123:665–671; http://dx.doi.org/10.1289/ehp.1408605

## Introduction

Humans experience ubiquitous exposure to phenols, including bisphenol A (BPA) and parabens. *The National Report on Human Exposure to Environmental Chemicals* [Centers for Disease Control and Prevention (CDC) 2013], which is periodically issued by the CDC and uses samples from the National Health and Nutrition Examination Survey (NHANES), detected BPA in 92.6% of a nationally representative sample in 2003–2004 (Calafat et al. 2008). Methyl and propyl paraben were detected in 99.1% and 92.7%, respectively, of 2005–2006 NHANES participants, and butyl paraben was detected in 40% of participants (Calafat et al. 2010), likely reflecting its less common use [Cosmetic Ingredient Review (CIR) Expert Panel 2008].

BPA is polymerized to manufacture polycarbonate plastic products (Biles et al. 1999), dental sealants (Olea et al. 1996), thermal receipt paper (Biedermann et al. 2010), and epoxy resin liners of some canned food containers (Braunrath et al. 2005). Parabens are used largely as antimicrobial preservatives in personal care products and pharmaceuticals (Guo and Kannan 2013). Ingestion is considered the main route of BPA exposure (Vandenberg et al. 2007), whereas dermal absorption is an important route of paraben exposure (CIR Expert Panel 2008). The half-life of BPA is approximately 6 hr, with nearly complete urinary excretion in 24 hr (Völkel et al. 2005); the urinary excretion of butyl paraben peaks 8–12 hr following dermal application (Janjua et al. 2008). Urinary concentrations of BPA and parabens can be used as biomarkers of their exposure (Calafat et al. 2010).

Rodent studies have linked phenols with adverse reproductive outcomes. BPA has been linked to increased oocyte aneuploidy (Hunt et al. 2003) and adverse effects on meiotic spindle formation (Can et al. 2005), centrosome dynamics (Lenie et al. 2008), and chromosome alignment and segregation (Machtinger et al. 2012). Both methyl and propyl parabens have been shown to affect mitochondrial activity (Nakagawa and Moldéus 1998; Prusakiewicz et al. 2007), which affects male fertility (Soni et al. 2002; Tavares et al. 2009), and they have also been shown in rats to bind estrogen receptors (Routledge et al. 1998).

Recent studies have shown that the paternal contribution to a healthy pregnancy is more important than previously thought. Beyond delivering their genome, sperm contribute physical structures necessary for fertilization (Cummins 2001; Sutovsky and Schatten 2000), as well as a variety of spermatozoal RNAs (Ostermeier et al. 2002, 2004, 2005). These RNAs are markers and potential effectors of human male infertility (Platts et al. 2007; Yatsenko et al. 2006; Zhao et al. 2007). Thus, paternal phenol exposure may impact sperm with potential implications for couples’ fertility.

The aim of this study was to examine associations of paternal urinary phenol concentrations (i.e., BPA and parabens) with fertilization rate, embryo quality, implantation, and live birth among couples from a fertility clinic.

## Methods

*Study participants and data collection*. This analysis is part of a larger prospective cohort study, the Environment and Reproductive Health (EARTH) Study, focused on environmental and nutritional determinants of fertility among couples from a fertility clinic (Braun et al. 2012; Smith et al. 2012). In 2004, the study began enrolling men 18–51 years and women 18–45 years of age, and at the time of this analysis consisted of 352 men and 579 women. Participants are followed from study entry until they have a live birth or discontinue treatment at the Massachusetts General Hospital (MGH) Fertility Center. All male–female couples whose male partner had phenols measured during his female partner’s cycle(s) in 2004–2012 were included. Up to three intrauterine insemination (IUI) and/or three fresh non-donor *in vitro* fertilization (IVF) cycles were included. Because a small percentage of men had cycles with missing phenol measurements (*n* = 18, 4.4%) that were excluded from the analysis, their included cycles were not necessarily consecutive. To account for the total number of treatment cycles each couple underwent, regardless of whether they were included in our analysis, we created a variable for total number of treatment cycles before excluding these cycles. Cycles converted from IUI to IVF or vice versa were excluded because their protocols differed from nonconverted cycles and there were too few cycles to analyze separately. All subjects provided informed consent, and this study was approved by the institutional review boards at the MGH Fertility Center, the Harvard T.H. Chan School of Public Health, and the CDC.

At recruitment, participants completed research nurse–administered and self-administered questionnaires regarding demographics, medical history, occupation, and lifestyle. Clinical information was obtained from the electronic medical record, and infertility diagnoses were classified according to the Society for Assisted Reproductive Technology (SART) definitions (Practice Committee of the American Society for Reproductive Medicine 2006).

*Treatment protocols, outcome, and exposure measurements*. Couples underwent IUI or IVF following an infertility evaluation. For IUI cycles, women underwent ovulation induction with clomiphene citrate or gonadotropins; cycles without medical induction were excluded because they differ from traditional IUI and there were too few to analyze separately. For IVF, women underwent one of the following three treatment protocols used at the MGH Fertility Center: *a*) luteal-phase GnRH (gonadotropin-releasing hormone)–agonist protocol using low, regular, and high-dose leuprolide (Lupron), in which pituitary desensitization was initiated in the luteal phase; *b*) follicular-phase GnRH-agonist/Flare protocol, in which Lupron was begun in the follicular phase on day 2 of menses; or *c*) GnRH-antagonist protocol, in which GnRH antagonist was initiated when the lead follicle reached 14 mm in size. All IVF cycles were preceded by a cycle of oral contraceptive pills unless contraindicated. On day 3 of menses, exogenous gonadotropins were initiated. Ovulation was induced with hCG (human chorionic gonadotropin) when at least three dominant follicles ≥ 16 mm were noted and peak estradiol level was > 600 pg/mL, and oocyte retrieval was performed approximately 36 hr later. Retrieved oocytes were fertilized by insemination or by intracytoplasmic sperm injection (ICSI), which was used for couples with severe male factor infertility and rarely for couples with prior failed fertilization. Embryos were evaluated by an embryologist and selected for transfer on day 2, 3, or 5 of maturation in culture.

For each cycle, men whose female partner was undergoing IVF or IUI provided one spot urine sample at the time of oocyte retrieval or insemination, respectively. Urine was collected in a clean polypropylene specimen cup, and specific gravity (SG) was measured at room temperature using a handheld refractometer (National Instrument Co. Inc., Baltimore, MD) calibrated with deionized water before each measurement. Urine samples were divided into aliquots, frozen, and stored at –80°C. Samples were shipped overnight on dry ice to the CDC, where they were stored at ≤ –40°C until blinded analysis. The urinary concentration of free plus conjugated phenol species (i.e., total concentration) was measured using online solid-phase extraction coupled to isotope dilution–high performance liquid chromatography–tandem mass spectrometry (Ye et al. 2005). Each analytical run included calibration standards, reagent blanks, and quality control materials. The limits of detection (LODs) for BPA and methyl, propyl, and butyl paraben were 0.36–0.40 ng/mL, 1.0 ng/mL, 0.2 ng/mL, and 0.2 ng/mL, respectively. Concentrations below the LOD were assigned a value of the LOD divided by the square root of two (Hornung and Reed 1990).

We considered the outcomes of fertilization rate, embryo quality, implantation, and live birth. Fertilization rate was defined as the number of oocytes with two pronuclei divided by the total number of mature metaphase II oocytes. Mature oocytes were those with a cumulus/corona complex at insemination; oocytes undergoing ICSI had polar body confirmation of the metaphase II phase after being denuded. Embryo quality was defined dichotomously as high-quality embryos versus lower quality embryos; high-quality embryos were those with four cells on culture day 2, eight cells on culture day 3, and high quality scores (taking into account symmetry and appearance of blastomeres and absence of fragmentation) on both culture days 2 and 3. Implantation was defined as a positive pregnancy test (βhCG ≥ 6 mIU/mL) 17 days after oocyte retrieval. Live birth was defined as the delivery of a live infant. Fertilization rate, embryo quality, and implantation were evaluated among couples who underwent IVF, and live birth was evaluated among all couples. IVF cycles with a day 2 embryo transfer were excluded from the models for implantation and live birth, because this generally indicates poor treatment response.

*Statistical analysis*. Some men provided multiple urine samples because their female partner underwent more than one IVF and/or IUI cycle. Therefore, the geometric means of SG-adjusted phenol concentrations from all measurements collected up to and including the cycle of interest were used to reflect phenol exposures. Multivariable generalized linear mixed models with exchangeable variance–covariance structures were used to evaluate associations of SG-adjusted paternal urinary phenol concentrations with fertilization rate, embryo quality, implantation, and live birth; all four of these outcomes were evaluated among IVF cycles, but only live birth was evaluated among IUI cycles. Embryo quality was modeled as the proportion of high-quality embryos among all embryos. For fertilization rate and embryo quality, estimates of the rate ratio (RR) were obtained for each quartile compared with the lowest quartile using a binomial distribution with a logit link. For implantation and live birth, estimates of the odds ratio (OR) were obtained for each quartile compared with the lowest quartile using a binary distribution with a logit link. Log-binomial models for the relative risk were attempted, but did not all converge, so the ORs are presented. BPA and methyl and propyl parabens were modeled as approximate quartiles. A linear trend test across quartiles was obtained by using the median of each quartile as a continuous variable. Because 67% of butyl paraben measurements were below the LOD, butyl paraben was modeled as undetectable versus detectable concentrations, and the *p*-value for the dichotomous variable is presented.

Potential confounders were based on prior literature and included overall cycle number, paternal and maternal age, paternal and maternal body mass index (BMI; normal weight vs. overweight/obese, overweight vs. normal weight/obese, and obese vs. normal weight/overweight), and paternal and maternal smoking (ever vs. never). Underweight, normal-weight, overweight, and obese were defined according to the Global Database of Body Mass Index (http://apps.who.int/bmi/) as BMI of < 18.5 kg/m^2^, 18.5–25 kg/m^2^, 25 to < 30 kg/m^2^, and ≥ 30 kg/m^2^, respectively. Although being underweight is associated with adverse reproductive outcomes (Ehrenberg et al. 2003), given their small number, underweight women were combined with normal-weight women. We considered the following as potential mediators of the relationship between paternal phenol concentration and IVF cycles outcomes: any diagnosis of male factor infertility, IVF treatment protocol (flare/antagonist vs. luteal phase), number of oocytes retrieved, use of ICSI, number of embryos transferred, and embryo transfer day. The only potential mediator considered for IUI cycles was any diagnosis of male-factor infertility. Potential confounders were evaluated in univariate models adjusted for maternal age at cycle start due to its biological relevance. The median urinary phenol concentration for each quartile was modeled as a continuous exposure, and potential confounders or mediators that changed the estimate for the male phenol concentration by 10% on the log odds scale and had a *p*-value < 0.2 were included in the adjusted models. Potential mediators were subsequently evaluated individually in models adjusting for confounders that met inclusion criteria for the model, as well as maternal age. Models are presented as unadjusted, adjusted for confounders, and adjusted for confounders and mediators. All analyses were conducted with SAS version 9.3 (SAS Institute Inc., Cary, NC), and two-sided *p*-values < 0.05 were considered to indicate statistically significant associations.

## Results

A total of 218 couples underwent 406 (195 IUI and 211 IVF) cycles. The number of first, second, and third IUI cycles were 102, 66, and 27, respectively; the numbers for IVF cycles were 152, 40, and 19, respectively. Fifty-three percent of couples underwent IVF only, 30% underwent IUI only, and 17% underwent IUI before undergoing IVF. Subsequent IUI cycles occurred approximately 4 weeks apart, whereas subsequent IVF cycles occurred approximately 16 weeks apart. At baseline, the mean ages of men and their female partners were 36.7 and 35.0 years, respectively (Table 1). Regarding primary SART diagnosis among the 218 couples, 32% had ovarian dysfunction, diminished ovarian reserve, or other female factor, 33% had male factor, and 35% had unexplained infertility. Thirty-nine percent of couples had either a primary or secondary diagnosis of male factor infertility. Two-thirds of women (67%) had a BMI < 25 kg/m^2^, and five (2.3%) were underweight. Among men, 30% were normal weight, and none were underweight (Table 1). Compared to the full cohort, our subsample of men was representative in terms of age, BMI, race, and smoking status (data not shown).

**Table 1:** Participant characteristics among 218 male–female couples at enrollment in the Environment and Reproductive Health (EARTH) Study.

Characteristic	Men	Women	Couples
Individual characteristic
Age (years)	36.7 ± 5.1	35.0 ± 4.0
< 37	118 (54.1)	150 (68.8)
≥ 37	100 (45.9)	68 (31.2)
BMI (kg/m^2^)	27.3 ± 4.1^*a*^	24.2 ± 4.5
< 25	65 (30.0)	146 (67.0)
25 to < 30	103 (47.5)	46 (21.1)
≥ 30	49 (22.6)	26 (11.9)
Race
White	183 (83.9)	178 (81.7)
Black/African American	5 (2.3)	3 (1.4)
Asian	16 (7.3)	20 (9.2)
Native American/Alaska Native	6 (2.8)	3 (1.4)
Other	7 (3.2)	14 (6.4)
Unknown	1 (0.5)	0 (0.0)
Smoking status
Never	145 (66.5)	156 (71.6)
Ever	73 (33.5)	62 (28.4)
Former (*n*)	60	55
Current (*n*)	13	7
Couple characteristic
Primary SART diagnosis at study entry
Male factor			71 (32.6)
Ovulatory dysfunction/DOR			41 (18.8)
Other female factor			29 (13.3)
Unexplained			77 (35.3)
Primary or secondary diagnosis of male factor infertility			85 (39.0)
Year of recruitment
2004–2006			41 (18.8)
2007–2009			102 (46.8)
2010–2012 (through April)			75 (34.4)
DOR, diminished ovarian reserve. Values are mean ± SD or *n* (%).^***a***^One value is missing.			

Cycles were nearly evenly split between IVF (52%) and IUI (48%) (Table 2). The majority of IVF cycles (70%) used luteal-phase stimulation, roughly half (56%) used ICSI, and 43% resulted in live birth. Among IUI cycles, 12% resulted in live birth, 79% resulted in no pregnancy, 8.2% resulted in pregnancy loss, which included chemical pregnancy, ectopic pregnancy, and spontaneous abortion, and 1.0% had an unknown outcome (Table 2).

**Table 2:** Cycle characteristics and pregnancy outcomes overall and by cycle^*a*^ among 218 couples at enrollment in the Environment and Reproductive Health (EARTH) Study.

Characteristic	All cycles	Cycle 1	Cycle 2	Cycle 3
*In vitro* fertilization (IVF)
No. of cycles	211	152	40	19
Protocol
Luteal phase	148 (70.1)	123 (80.9)	19 (47.5)	6 (31.6)
Flare	39 (18.5)	16 (10.5)	14 (35.0)	9 (47.4)
Antagonist	24 (11.4)	13 (8.6)	7 (17.5)	4 (21.1)
Intracytoplasmic sperm injection	119 (56.4)	80 (52.6)	26 (65.0)	13 (68.4)
Oocytes retrieved	11.0 ± 5.2	11.3 ± 5.3	10.6 ± 5.2	9.0 ± 3.7
Metaphase II oocytes retrieved	9.3 ± 4.6	9.6 ± 4.5	9.1 ± 5.0	7.6 ± 3.7
Day of embryo transfer^*b,c*^
2	11 (5.5)	9 (6.2)	2 (5.3)	0 (0.0)
3	124 (61.4)	81 (55.9)	25 (65.8)	18 (94.7)
5	67 (33.2)	55 (37.9)	11 (29.0)	1 (5.3)
No. of embryos transferred^*c*^
1	30 (14.8)	24 (16.4)	6 (13.2)	1 (5.3)
2	130 (64.0)	97 (66.4)	23 (60.5)	10 (52.6)
≥ 3	43 (21.2)	25 (17.1)	10 (26.3)	8 (42.1)
Pregnancy outcome^*d*^
No transfer	8 (3.8)	6 (4.0)	2 (5.0)	0 (0.0)
No oocytes retrieved	3	2	1	—
Fertilization failure	5	4	1	—
Implantation failure^*c*^	78 (38.4)	47 (32.2)	20 (52.6)	11 (57.9)
Chemical pregnancy^*c*^	13 (6.4)	9 (6.2)	3 (7.8)	1 (5.3)
Ectopic pregnancy^*c*^	3 (1.4)	3 (2.0)	0 (0.0)	0 (0.0)
Spontaneous abortion^*c*^	19 (9.0)	17 (11.2)	1 (2.5)	1 (5.3)
Therapeutic abortion^*c*^	1 (0.5)	1 (0.7)	0 (0.0)	0 (0.0)
Stillbirth^*c*^	1 (0.5)	0 (0.0)	1 (2.5)	0 (0.0)
Live birth^*c*^	88 (43.4)	69 (47.3)	13 (34.2)	6 (31.6)
Intrauterine insemination (IUI)
No. of cycles	195	102	66	27
Pregnancy outcome^*c*^
Not pregnant^*e*^	153 (78.5)	79 (77.5)	51 (77.3)	24 (85.7)
Chemical pregnancy	5 (2.6)	4 (3.9)	0 (0.0)	1 (3.7)
Ectopic pregnancy	1 (0.5)	1 (1.0)	0 (0.0)	0 (0.0)
Spontaneous abortion	10 (5.1)	6 (5.9)	2 (3.0)	2 (7.4)
Live birth	24 (12.2)	11 (10.8)	12 (18.2)	1 (3.7)
Unknown	2 (1.0)	1 (1.0)	1 (1.5)	0 (0.0)
Values are *n* (%) or mean ± SD.^***a***^IVF cycle was defined as the consecutive IVF cycle number among all IVF cycles included in the analysis; IUI cycle was defined as the consecutive IUI cycle number among all IUI cycles included in the analysis. ^***b***^One missing value. ^***c***^Calculated among cycles with an embryo transfer. ^***d***^Implantation was defined as a positive pregnancy test (βhCG ≥ 6 mIU/mL) 17 days following embryo transfer; chemical pregnancy was defined as implantation with no subsequent clinical pregnancy; ectopic pregnancy was defined as a pregnancy outside the uterus; spontaneous abortion was defined as spontaneous loss of a clinical pregnancy; therapeutic abortion was defined as induced abortion of a clinical pregnancy; stillbirth was defined as the delivery of a dead infant; live birth was defined as delivery of a live infant. ^***e***^Not pregnant was defined as a negative pregnancy test (βhCG < 6 mIU/mL) 17 days following intrauterine insemination; chemical pregnancy was defined as a positive pregnancy test (βhCG ≥ 6 mIU/mL) 17 days following insemination; ectopic pregnancy, spontaneous abortion, and live birth defined as described above.				

The median geometric mean of paternal unadjusted BPA among all IVF cycles in our analysis was 1.6 ng/mL [interquartile range (IQR) = 0.8–2.8 ng/mL], which was slightly lower than the concentrations measured in males in NHANES 2007–2008 (2.20 ng/mL) and 2009–2010 (1.94 ng/mL), though these males include children and older men (CDC 2013). The median geometric mean concentrations of unadjusted paternal methyl and propyl paraben among all IVF cycles in our analysis were 26.1 ng/mL (IQR = 10.3–87.5 ng/mL) and 3.4 ng/mL (IQR = 0.8–15.1 ng/mL), respectively. Although these methyl paraben concentrations were slightly lower than those among males in NHANES 2007–2008 (33.6 ng/mL) and 2009–2010 (31.7 ng/mL), the concentrations of propyl paraben were slightly higher (3.02 ng/mL and 2.77 ng/mL, respectively) (CDC 2013). Among all IVF cycles in our analysis, 60% of paternal urinary butyl paraben measurements were below the LOD (< 0.2 ng/mL), which was similar to NHANES (CDC 2013). For both IVF and IUI cycles, at each cycle number, the medians of the single measurements taken at that cycle (“concurrent measures”) across all men in the analysis were similar to the median geometric means of concentrations measured up to and including that cycle. Additionally, the median paternal SG-adjusted geometric means of the phenols among all IUI cycles in our analysis were similar to those from IVF cycles (see Supplemental Material, Table S1).

*Covariates associated with IUI and IVF outcomes*. Given univariate models to identify covariates that were associated with the outcomes at the α = 0.20 level and that produced ≥ 10% change in the log odds, similar to other studies, each additional year of maternal age was associated with decreased odds of live birth following IVF [OR = 0.93; 95% confidence interval (CI): 0.86, 1.01] and IUI (OR = 0.92; 95% CI: 0.82, 1.02). Each additional year of paternal age was marginally associated with decreased odds of implantation (OR = 0.95; 95% CI: 0.89, 1.02) and live birth following IVF (OR = 0.95; 95% CI: 0.89, 1.01), whereas maternal and paternal normal weight were negatively associated with the proportion of high-quality embryos compared with participants who were overweight or obese (RR = 0.58; 95% CI: 0.38, 0.90 and RR = 0.65; 95% CI: 0.41, 1.02, respectively). Each additional cycle number was negatively associated with implantation (OR = 0.79; 95% CI: 0.65, 0.96) and live birth following IVF (OR = 0.86; 95% CI: 0.71, 1.04), because couples requiring more cycles are more likely to have lower fertility. Similarly, maternal ever smoking compared with never smoking was associated with lower proportions of high-quality embryos (RR = 0.73; 95% CI: 0.46, 1.17) and decreased odds of implantation (OR = 0.53; 95% CI: 0.26, 1.08) and live birth following IVF (OR = 0.61; 95% CI: 0.31, 1.21). The use of ICSI was associated with lower odds of implantation (OR = 0.59; 95% CI: 0.31, 1.13), likely because couples using ICSI have poorer fertility, as reflected by prior failures of IVF without ICSI. Live birth following IVF was positively associated with luteal-phase stimulation protocol (OR = 1.85; 95% CI: 0.95, 3.60), a greater number of oocytes retrieved (OR = 1.04; 95% CI: 0.98, 1.10), and day 5 embryo transfer; (OR = 1.86; 95% CI: 0.98, 3.52), but it was negatively associated with a greater number of embryos transferred (OR = 0.70; 95% CI: 0.41, 1.18).

*Paternal urinary phenol concentrations and IUI and IVF outcomes*. Paternal urinary phenol concentrations were not associated with fertilization rate (see Supplemental Material, Table S2), which was evaluated only in IVF cycles, or live birth following IVF (Table 3) in either unadjusted or adjusted models. After adjustment for confounders, male BPA concentrations in the second quartile were associated with a greater proportion of high-quality embryos [adjusted RR (aRR) = 1.92; 95% CI: 1.13, 3.25] in IVF cycles, but higher quartiles showed no association, and no associations were seen with paternal urinary paraben concentrations (see Supplemental Material, Table S3). After adjustment for confounders and mediators, paternal phenol concentrations were not significantly associated with odds of implantation, which was evaluated in IVF cycles (see Supplemental Material, Table S4). After adjusting for confounders, paternal methyl paraben concentrations in the second quartile were associated with decreased odds of live birth following IUI (aOR = 0.19; 95% CI: 0.04, 0.82), and propyl paraben concentrations in the fourth quartile were suggestive of decreased odds (OR = 0.20; 95% CI: 0.04, 1.02; *p* = 0.053; Table 4).

**Table 3:** Associations between live birth and quartiles of specific gravity–adjusted urinary phenol concentrations among men whose partner underwent *in vitro* fertilization.

Phenol quartiles(range in ng/mL)	Live births/cycles	Unadjusted OR (95% CI)	Confounder-adjusted^*a*^ aOR (95% CI)	Confounder- and mediator-adjusted^*b*^ aOR (95% CI)
BPA	87/200
1 (≤ 1.05)	20/50	1.00 (reference)	1.00 (reference)	1.00 (reference)
2 (1.06–1.81)	22/51	1.16 (0.50, 2.73)	1.15 (0.49, 2.69)	0.88 (0.36, 2.13)
3 (1.82–2.51)	17/49	0.80 (0.33, 1.94)	0.82 (0.34, 1.97)	0.69 (0.28, 1.72)
4 (2.52–22.91)	28/50	1.92 (0.81, 4.53)	2.04 (0.86, 4.83)	1.65 (0.67, 4.05)
*p*-Trend^*c*^		0.11	0.08	0.16
Methyl paraben	83/189
1 (≤ 10.46)	23/48	1.00 (reference)	1.00 (reference)	1.00 (reference)
2 (10.47–28.95)	17/46	0.67 (0.27, 1.64)	0.91 (0.36, 2.30)	0.72 (0.28, 1.89)
3 (28.96–109)	24/49	1.04 (0.44, 2.49)	1.16 (0.49, 2.78)	1.20 (0.49, 2.97)
4 (109–2,862)	19/46	0.77 (0.32, 1.89)	0.82 (0.34, 1.98)	0.74 (0.30, 1.81)
*p*-Trend^*c*^		0.76	0.61	0.55
Propyl paraben	83/189
1 (≤ 0.99)	23/48	1.00 (reference)	1.00 (reference)	1.00 (reference)
2 (1.00–3.47)	19/45	0.79 (0.32, 1.96)	0.91 (0.37, 2.25)	1.14 (0.44, 2.95)
3 (3.48–13.93)	20/50	0.70 (0.29, 1.70)	0.78 (0.32, 1.92)	0.95 (0.37, 2.44)
4 (13.94–842)	21/46	0.90 (0.36, 2.23)	0.92 (0.38, 2.26)	1.06 (0.42, 2.67)
*p*-Trend^*c*^		0.85	0.98	0.94
Butyl paraben	83/189
Below the LOD^*d*^	52/115	1.00 (reference)	1.00 (reference)	1.00 (reference)
Above the LOD^*d*^	31/74	0.85 (0.45, 1.62)	0.85 (0.44, 1.67)	0.86 (0.44, 1.67)
*p*-Value^*e*^		0.62	0.64	0.64
aOR, adjusted odds ratio. ^***a***^All models were adjusted for maternal age (years); the models for methyl and propyl paraben were also adjusted for overall cycle number; the models for propyl and butyl paraben were also adjusted for maternal smoking (ever vs. never). ^***b***^All models were adjusted for all variables listed above and embryo transfer day; the model for propyl paraben was also adjusted for any diagnosis of male factor infertility (yes vs. no); models for methyl, propyl, and butyl paraben were also adjusted for stimulation protocol (luteal phase vs. flare/antagonist); the model for methyl paraben was also adjusted for use of ICSI (yes vs. no). ^***c***^The *p*-trend value was calculated using the median of each quartile as a continuous variable. ^***d***^The LOD for butyl paraben was 0.2 ng/mL. ^***e***^The *p*-value was calculated using above and below the LOD as categorical variables.				

**Table 4:** Associations between live birth among initiated cycles and quartiles of specific gravity–adjusted geometric mean urinary phenol concentrations among men whose partner underwent intrauterine insemination.

Phenol quartiles (range in ng/mL)	Live births/cycles	Unadjusted OR (95% CI)
BPA	24/195
1 (≤ 1.16)	8/48	1.00 (reference)	1.00 (reference)
2 (1.17–1.82)	8/49	0.99 (0.32, 3.03)	0.95 (0.31, 2.93)
3 (1.83–2.96)	2/49	0.21 (0.04, 1.11)	0.24 (0.04, 1.25)
4 (2.97–32.97)	6/49	0.69 (0.21, 2.28)	0.66 (0.20, 2.21)
*p*-Trend^*b*^		0.43	0.41
Methyl paraben	24/182
1 (≤ 10.66)	10/45	1.00 (reference)	1.00 (reference)
2 (10.67–25.15)	3/46	0.25 (0.06, 0.99)	0.19 (0.04, 0.82)
3 (25.16–78.17)	5/46	0.43 (0.13, 1.42)	0.34 (0.09, 1.21)
4 (79–4,687)	6/45	0.54 (0.17, 1.70)	0.35 (0.10, 1.25)
*p*-Trend^*b*^		0.92	0.58
Propyl paraben	24/182
1 (≤ 0.90)	7/46	1.00 (reference)	1.00 (reference)
2 (0.91–2.66)	7/45	1.04 (0.32, 3.40)	0.78 (0.22, 2.75)
3 (2.67–11.37)	7/45	1.03 (0.31, 3.42)	0.90 (0.26, 3.14)
4 (11.38–981)	3/46	0.38 (0.09, 1.68)	0.20 (0.04, 1.02)
*p*-Trend^*b*^		0.14	0.04
Butyl paraben	24/182
Below the LOD^*c*^	15/122	1.00 (reference)	1.00 (reference)
Above the LOD^*c*^	9/60	1.27 (0.51, 3.18)	1.18 (0.47, 2.97)
*p*-Value^*d*^		0.61	0.73
aOR, adjusted odds ratio. ^***a***^All models were adjusted for maternal age (years); maternal smoking (ever vs. never) was additionally adjusted for in the models for methyl and propyl paraben. ^***b***^The *p*-trend value was calculated using the median of each quartile as a continuous variable. ^***c***^The LOD for butyl paraben was 0.2 ng/mL. ^***d***^The *p*-value was calculated using above and below the LOD as categorical variables. No mediator met the inclusion criteria for any model, so we present a mode adjusted for confounders only.			

## Discussion

In this analysis we examined the association of paternal urinary phenol concentrations with couple-level reproductive outcomes. Prior work in this cohort among women undergoing IVF showed that higher maternal urinary BPA concentrations were associated with decreased ovarian response (Mok-Lin et al. 2010), decreased peak serum estradiol, fewer oocytes retrieved, fewer normally fertilized oocytes, and reduced implantation (Ehrlich et al. 2012a, 2012b). In this analysis, paternal concentrations of methyl paraben in the second quartile and propyl paraben in the fourth quartile were significantly associated with and suggestive of decreased odds of live birth following IUI, respectively. However, because there were only three, five, and six live births in the second, third, and fourth quartiles of methyl paraben, respectively, and there were only three live births in the fourth quartile of propyl paraben, these estimates should be interpreted with caution. These results do not clearly indicate or preclude adverse effects of paternal exposure to environmental phenols on reproductive outcomes, and much research remains to further elucidate these potential effects.

It is interesting to note the differences found between IUI and IVF, with null results in IVF cycles and significant and suggestive associations for specific quartiles of methyl and propyl paraben, respectively, in IUI cycles. One possible explanation may be the differing invasiveness of the procedures. For instance, if phenols adversely affect reproductive outcomes, the intensive interventions of IVF may overcome these effects, whereas the less intense IUI may not.

To our knowledge, there are few if any studies in experimental animals on pregnancy outcomes following paternal exposure to phenols, but there is growing evidence of paternally mediated adverse health effects of phenols. In one study in which adult male rats, used as sires, were chronically exposed to 50 μg/kg/day of dietary BPA, the adult F_1_ offspring had decreased acquisition and retention of spatial memory compared with unexposed F_1_ offspring (Fan et al. 2013). In another study in mice, maternal exposure to plastics-derived endocrine disruptors, including BPA, promoted epigenetic transgenerational inheritance of adult disease mediated through the male germline (Manikkam et al. 2013). Finally, in male offspring exposed during gestation to a mixture of BPA and phthalates, differential DNA methylation of the F_3_ generation sperm promoter epigenome was identified (Manikkam et al. 2012).

There are few epidemiologic studies on male-mediated effects of phenols. We reported an association of urinary BPA with altered semen quality, as evidenced by declines of 23% in sperm concentration, 8% in motility, and 13% in morphology in a cross-sectional study of 190 men from the MGH Fertility Center (Meeker et al. 2010). Poorer semen parameters were also associated with increased urinary BPA concentrations in a study of occupationally exposed Chinese workers (Li et al. 2011), and a modest reduction in free testosterone was found among 375 fertile men (Mendiola et al. 2010). A small study, similar in design to ours, of 27 couples undergoing IVF found inverse associations between paternal concentrations of serum unconjugated BPA measured on the day of oocyte retrieval and altered embryo cell number and increased embryo fragmentation score (Bloom et al. 2011). Urinary paraben concentrations have also been associated with sperm DNA damage in male partners from the MGH Fertility Center (Meeker et al. 2011).

*Strengths and limitations*. To our knowledge, this study is one of the first to examine associations of paternal preconception urinary concentrations of BPA and parabens with pregnancy and early reproductive outcomes. Another couple-based prospective cohort study with preconception enrollment, the Longitudinal Investigation of Fertility and the Environment (LIFE) Study, has reported associations between decreased couple fecundity and maternal and paternal exposure to environmental chemicals (Buck Louis et al. 2012, 2013). The authors found no association between maternal or paternal urinary BPA concentrations and time to pregnancy (Buck Louis et al. 2014). Our study examined early reproductive outcomes among couples undergoing assisted reproduction, and though these couples differ from the general population, they may be a population sensitive to these exposures. Our subsample of men was representative of the full cohort with respect to age, BMI, race, and smoking status. This subsample excluded men who participated without their female partner and men whose urine sample had not yet been analyzed, but these reasons are unlikely to introduce any bias.

A limitation is potential exposure misclassification due to the short half-life of phenols, which can result in high within-individual variability. The intraclass correlation coefficient (ICC) of BPA has been shown to be as low as 0.12 (Braun et al. 2012), though parabens appear to be less variable, particularly among men (ICC = 0.54 and ICC = 0.51 for methyl and propyl paraben, respectively) (Smith et al. 2012). Low ICCs can reduce study power and increase the probability of type 2 error. In our study, the geometric means were similar to concurrent measurements, though we believe using the geometric mean concentration of all samples collected before the cycle minimized this potential bias. Any residual misclassification should be nondifferential, though due to the use of categorical exposure, this could result in bias toward or away from the null. Finally, we adjusted for variables that may be mediators as opposed to confounders. For instance, if phenol exposures adversely affect reproduction, they could reduce the number of viable oocytes available for retrieval, which could also decrease the number of embryos available for transfer, which in turn may affect implantation. When controlling for a mediator, it is possible that the total causal effect of the exposure on the outcome is not consistently estimated; typically, this will bias results toward the null (Schisterman et al. 2009). Thus, these estimates may underestimate the true causal effect. Additionally, race and ethnicity have been shown to be determinants of exposure, particularly for parabens (Calafat et al. 2010). As our population was 83% Caucasian, these phenol concentrations may not be representative of population concentrations.

## Conclusions

This study does not exclude the possibility of adverse effects of male phenol exposure on reproductive outcomes in couples undergoing assisted reproduction. Further understanding of these associations could result from replication within a larger and more diverse cohort, and investigations into exposure to mixtures of environmental chemicals, within both individuals and couples, would further elucidate the reproductive effects of these exposures.

## Supplemental Material

(316 KB) PDF
