# Supplementary material for: Paternal Urinary Concentrations of Parabens and Other Phenols in Relation to Reproductive Outcomes among Couples from a Fertility Clinic
Source: Environ Health Perspect. 2015 Mar 13;123(7):665–71. doi: 10.1289/ehp.1408605 (PMC4492268; doi:10.1289/ehp.1408605)
Supplement: (316 KB) PDF [file ehp.1408605.s001.acco.pdf]

Environ Health Perspect
doi: 10.1289/ehp.1408605

Note to Readers: ***EHP*** **strives to ensure that all journal content is accessible to all readers. However, some figures and Supplemental Material published in** ***EHP*** **articles may not conform to 508 standards due to the complexity of the information being presented. If you need assistance accessing journal content, please contact ehp508@niehs.nih.gov. Our staff will work with you to assess and meet your accessibility needs within 3 working days.**

Supplemental Material

Paternal Urinary Concentrations of Parabens and Other Phenols in Relation to Reproductive Outcomes among Couples from a Fertility Clinic

Laura E. Dodge, Paige L. Williams, Michelle A. Williams, Stacey A. Missmer, Thomas L. Toth, Antonia M. Calafat, and Russ Hauser

Table of Contents

Table S1. Distribution of unadjusted and specific gravity (SG)-adjusted paternal urinary phenol concentrations (ng/ml).

Table S2. Associations between fertilization rate among initiated cycles and quartiles of geometric means of specific gravity-adjusted urinary phenol concentrations among men whose female partner underwent in vitro fertilization.

Table S3. Associations between the proportion of high-quality embryos among initiated cycles and quartiles of specific gravity-adjusted geometric mean urinary phenol concentrations among men whose female partner underwent in vitro fertilization.

Table S4. Associations between implantation and quartiles of geometric means of specific gravity-adjusted urinary phenol concentrations among men whose female partner underwent in vitro fertilization.

Table S1. Distribution of unadjusted and specific gravity (SG)-adjusted paternal urinary phenol concentrations (ng/ml).

Concentration	All cycles	Cycle 1	Cycle 2	Cycle 3
**In vitro fertilization (IVF)**				
Bisphenol A				
Number of cycles	211	152	40	19
Unadjusted geometric mean	1.6 (0.8 – 2.8)	1.9 (0.7 – 3.1)	1.3 (0.8 – 2.5)	1.2 (0.6 – 2.0)
SG-adjusted geometric mean	1.8 (1.1 – 2.5)	1.9 (1.1 – 2.7)	1.8 (1.1 – 2.4)	1.7 (1.0 – 2.1)
SG-adjusted concurrent measure	1.8 (1.1 – 2.7)	1.8 (1.0 – 2.8)	1.6 (1.1 – 2.5)	2.2 (1.5 – 2.9)
<LOD[a]—n (%)	30 (14.2)	22 (14.5)	5 (12.5)	3 (15.8)
Methyl paraben				
Number of cycles	199	143	38	18
Unadjusted geometric mean	26.1 (10.3 – 87.5)	24.9 (9.5 – 105.0)	30.7 (11.4 – 81.9)	29.0 (10.3 – 70.6)
SG-adjusted geometric mean	29.3 (10.7 – 108.6)	29.3 (8.9 – 108.6)	31.4 (13.3 – 122.0)	27.7 (16.2 – 94.1)
SG-adjusted concurrent measure	28.5 (8.3 – 122.3)	27.8 (8.1 – 122.3)	34.4 (14.4 – 101.7)	35.2 (7.3 – 139.4)
<LOD[a]—n (%)	2 (1.0)	1 (0.7)	1 (2.6)	0 (0.0)
Propyl paraben				
Number of cycles	199	143	38	18
Unadjusted geometric mean	3.4 (0.8 – 15.1)	3.1 (0.8 – 16.0)	4.1 (0.8 – 12.4)	3.3 (1.0 – 8.9)
SG-adjusted geometric mean	3.5 (1.0 – 13.9)	3.3 (0.8 – 13.9)	4.9 (1.0 – 19.6)	2.8 (1.5 – 12.9)
SG-adjusted concurrent measure	3.3 (0.7 – 20.2)	2.9 (0.7 – 20.2)	7.4 (0.7 – 47.4)	1.9 (0.7 – 9.6)
<LOD[a]—n (%)	23 (11.6)	18 (12.6)	3 (7.9)	2 (11.1)
Butyl paraben				
Number of cycles	199	143	38	18
Unadjusted geometric mean	0.2 (0.2 – 0.7)	0.2 (0.2 – 0.7)	0.2 (0.2 – 0.8)	0.2 (0.2 – 0.5)
SG-adjusted geometric mean	0.4 (0.2 – 1.0)	0.3 (0.2 – 1.0)	0.4 (0.2 – 0.9)	0.3 (0.2 – 0.5)
SG-adjusted concurrent measure	0.3 (0.2 – 0.8)	0.4 (0.2 – 0.9)	0.4 (0.1 – 0.7)	0.2 (0.2 – 0.6)
<LOD[a]—n (%)	119 (59.8)	82 (57.3)	25 (65.8)	12 (66.7)
**Intrauterine insemination (IUI)**				
Bisphenol A				
Number of cycles	195	102	66	27
Unadjusted geometric mean	2.0 (1.0 – 3.6)	1.8 (0.9 – 3.6)	2.1 (1.0 – 3.3)	1.8 (1.0 – 3.9)
SG-adjusted geometric mean	1.9 (1.2 – 3.0)	2.0 (1.0 – 3.0)	1.9 (1.2 – 3.1)	1.5 (1.3 – 2.9)
SG-adjusted concurrent measure	1.8 (1.1 – 3.0)	2.0 (1.0 – 3.0)	2.1 (1.3 – 3.2)	1.6 (1.0 – 2.4)
<LOD[a]—n (%)	18 (9.2)	11 (10.8)	5 (7.6)	2 (7.4)

Concentration	All cycles	Cycle 1	Cycle 2	Cycle 3
Methyl paraben				
Number of cycles	182	96	62	25
Unadjusted geometric mean	24.9 (9.3 – 85.6)	22.7 (8.2 – 84.8)	26.9 (10.0 – 101.9)	24.4 (13.3 – 92.9)
SG-adjusted geometric mean	25.5 (10.9 – 78.2)	21.8 (9.4 – 80.0)	29.1 (11.5 – 80.8)	32.5 (13.8 – 61.7)
SG-adjusted concurrent measure	23.1 (9.4 – 78.3)	21.8 (9.4 – 80.0)	20.2 (9.4 – 73.3)	31.2 (9.6 – 145.4)
<LOD[a]—n (%)	2 (1.1)	2 (2.1)	0 (0.0)	0 (0.0)
Propyl paraben				
Number of cycles	182	96	62	25
Unadjusted geometric mean	2.7 (0.9 – 11.3)	2.4 (0.9 – 9.7)	3.3 (0.9 – 11.6)	3.2 (1.6 – 15.0)
SG-adjusted geometric mean	2.7 (0.9 – 15.5)	2.6 (0.9 – 12.2)	3.9 (0.9 – 11.4)	2.9 (1.3 – 19.0)
SG-adjusted concurrent measure	2.7 (0.8 – 19.4)	2.6 (0.9 – 12.2)	2.8 (0.7 – 21.6)	8.5 (0.8 – 26.7)
<LOD[a]—n (%)	17 (9.3)	9 (9.4)	5 (8.1)	3 (12.5)
Butyl paraben				
Number of cycles	182	96	62	25
Unadjusted geometric mean	0.2 (0.2 – 0.3)	0.2 (0.2 – 0.3)	0.2 (0.2 – 0.4)	0.2 (0.2 – 0.7)
SG-adjusted geometric mean	0.2 (0.2 – 0.6)	0.2 (0.2 – 0.5)	0.2 (0.2 – 0.5)	0.3 (0.2 – 0.6)
SG-adjusted concurrent measure	0.2 (0.2 – 0.6)	0.2 (0.2 – 0.5)	0.2 (0.2 – 0.6)	0.2 (0.1 – 0.5)
<LOD[a]—n (%)	122 (66.7)	64 (66.7)	42 (67.7)	16 (66.7)

Data are presented as median and interquartile range of the geometric mean of all urinary phenol measurements up to and including the cycle of interest; IVF, in vitro fertilization; LOD, limit of detection; IUI, intrauterine insemination

[a]The LOD for bisphenol A was 0.36-0.40 ng/ml, the LOD for methyl paraben was 1.0 ng/ml, and the LODs for propyl and butyl paraben were 0.2 ng/ml

Table S2. Associations between fertilization rate among initiated cycles and quartiles of geometric means of specific gravity-adjusted urinary phenol concentrations among men whose female partner underwent in vitro fertilization.

Phenol quartiles (range in ng/ml)	N	Unadjusted RR (95% CI)	Confounder-adjusted[a] aRR (95% CI)
**Bisphenol A**	211		
1 (≤1.05)	53	1.00	1.00
2 (1.06 – 1.81)	53	1.37 (0.91, 2.06)	1.37 (0.90, 2.06)
3 (1.82 – 2.52)	52	1.24 (0.82, 1.88)	1.24 (0.82, 1.89)
4 (2.53 – 22.91)	53	1.20 (0.79, 1.82)	1.20 (0.79, 1.82)
**P-trend[b]**		0.72	0.72
**Methyl paraben**	199		
1 (≤10.46)	51	1.00	1.00
2 (10.47 – 28.95)	48	1.18 (0.78, 1.79)	1.18 (0.77, 1.80)
3 (28.96 – 109)	51	1.46 (0.95, 2.25)	1.46 (0.95, 2.25)
4 (109 – 2,862)	49	0.99 (0.64, 1.54)	0.99 (0.64, 1.55)
**P-trend[b]**		0.51	0.52
**Propyl paraben**	199		
1 (≤0.99)	50	1.00	1.00
2 (1.00 – 3.47)	49	1.13 (0.74, 1.74)	1.13 (0.74, 1.75)
3 (3.48 – 13.93)	51	1.07 (0.70, 1.64)	1.07 (0.69, 1.64)
4 (13.94 – 842)	49	0.99 (0.64, 1.53)	0.99 (0.64, 1.53)
**P-trend[b]**		0.68	0.69
**Butyl paraben**	199		
Below the LOD[c]	119	1.00	1.00
Above the LOD[c]	80	1.00 (0.75, 1.34)	1.00 (0.75, 1.35)
**P[d]**		0.97	0.98

Fertilization rate was defined as the number of oocytes with two pronuclei divided by the number of mature metaphase II oocytes; IVF, in vitro fertilization; RR, rate ratio; CI, confidence interval; LOD, limit of detection.

[a]All models were adjusted for maternal age (years); no other confounders met the inclusion criteria for any model. [b]The p-trend value was calculated using the median of each quartile as a continuous variable. [c]The LOD for butyl paraben was 0.2 ng/ml. [d]The p-value was calculated using above and below the LOD as categorical variables.

No mediators met the inclusion criteria for any model, therefore we present a model adjusted for confounders only.

Table S3. Associations between the proportion of high-quality embryos among initiated cycles and quartiles of specific gravity-adjusted geometric mean urinary phenol concentrations among men whose female partner underwent in vitro fertilization.

Phenol quartiles (range in ng/ml)	N	Unadjusted RR (95% CI)	Confounder-adjusted[a] aRR (95% CI)
**Bisphenol A**	211		
1 (≤1.05)	53	1.00	1.00
2 (1.06–1.81)	53	1.91 (1.12, 3.25)	1.92 (1.13, 3.25)
3 (1.82–2.52)	52	1.31 (0.76, 2.28)	1.36 (0.79, 2.36)
4 (2.53–22.91)	53	1.24 (0.71, 2.15)	1.26 (0.73, 2.18)
**P-trend[b]**		0.80	0.85
**Methyl paraben**	199		
1 (≤10.46)	49	1.00	1.00
2 (10.47–28.95)	50	1.20 (0.70, 2.08)	1.14 (0.66, 1.98)
3 (28.96–109)	51	1.33 (0.76, 2.33)	1.34 (0.76, 2.34)
4 (109–2,862)	49	1.05 (0.58, 1.91)	1.08 (0.60, 1.95)
**P-trend[b]**		0.79	0.94
**Propyl paraben**	199		
1 (≤0.99)	51	1.00	1.00
2 (1.00–3.47)	48	1.14 (0.65, 2.01)	1.16 (0.66, 2.04)
3 (3.48–13.93)	51	0.75 (0.44, 1.32)	0.77 (0.44, 1.35)
4 (13.94–842)	49	0.99 (0.55, 1.79)	1.10 (0.61, 1.98)
**P-trend**		0.90	0.64
**Butyl paraben**	199		
Below the LOD[c]	119	1.00	1.00
Above the LOD[c]	80	1.22 (0.84, 1.78)	1.25 (0.85, 1.82)
**P[d]**		0.29	0.25

RR, rate ratio; aRR, adjusted rate ratio; CI, confidence interval; LOD, limit of detection.

[a]All models were adjusted for maternal age (years) and paternal normal weight (vs. overweight and obese); maternal normal weight (vs. overweight and obese) was additionally adjusted for in the models for bisphenol A and methyl and propyl paraben; maternal smoking (ever vs. never) was additionally adjusted for in the model for bisphenol A. [b]The p-trend value was calculated using the median of each quartile as a continuous variable. [c]The LOD for butyl paraben was 0.2 ng/ml. [d]The p-value was calculated using above and below the LOD as categorical variables.

No mediator met the inclusion criteria for any model, therefore we present a model adjusted for confounders only.

Table S4. Associations between implantation and quartiles of geometric means of specific gravity-adjusted urinary phenol concentrations among men whose female partner underwent in vitro fertilization.

Phenol quartiles (range in ng/ml)	N	Unadjusted OR (95% CI)	Confounder-adjusted[a] aOR (95% CI)	Confounder- and mediator-adjusted[b] aOR (95% CI)
**Bisphenol A**	192			
1 (≤1.05)	43	1.00	1.00	NA[d]
2 (1.06 – 1.81)	51	1.03 (0.41, 2.57)	1.05 (0.41, 2.69)	NA[d]
3 (1.82 – 2.51)	49	1.02 (0.40, 2.61)	1.02 (0.39, 2.68)	NA[d]
4 (2.52 – 22.91)	49	2.06 (0.77, 5.51)	2.37 (0.85, 6.61)	NA[d]
**P-trend[c]**		0.11	0.07	NA[d]
**Methyl paraben**	182			
1 (≤10.46)	47	1.00	1.00	1.00
2 (10.47 – 29.38)	45	0.31 (0.12, 0.84)	0.42 (0.14, 1.26)	0.38 (0.13, 1.16)
3 (29.39 – 109)	44	0.72 (0.26, 1.98)	0.86 (0.29, 2.53)	0.86 (0.29, 2.54)
4 (109 – 2,862)	46	0.50 (0.18, 1.36)	0.55 (0.19, 1.61)	0.53 (0.18, 1.55)
**P-trend[c]**		0.70	0.59	0.57
**Propyl paraben**	182			
1 (≤0.99)	46	1.00	1.00	1.00
2 (1.00 – 3.47)	46	0.81 (0.30, 2.19)	1.02 (0.36, 2.87)	1.11 (0.37, 3.27)
3 (3.48 – 13.93)	44	0.63 (0.24, 1.62)	0.77 (0.28, 2.11)	0.76 (0.26, 2.17)
4 (13.94 – 842)	46	1.01 (0.37, 2.75)	0.95 (0.34, 2.67)	0.98 (0.33, 2.95)
**P-trend[c]**		0.62	0.98	0.95
**Butyl paraben**	182			
Below the LOD[e]	112	1.00	1.00	1.00
Above the LOD[e]	70	0.69 (0.35, 1.36)	0.75 (0.36, 1.57)	0.67 (0.31, 1.42)
**P[f]**		0.28	0.44	0.29

OR, odds ratio; aOR, adjusted odds ratio; CI, confidence interval; LOD, limit of detection.

[a]All models are adjusted for maternal age (years) and overall cycle number; paternal age (years) was additionally adjusted for in the models for methyl and butyl paraben; maternal smoking (ever vs. never) was additionally adjusted for in the models for methyl, propyl, and butyl paraben. [b]All models were adjusted for all variables listed above; any diagnosis of male factor infertility (yes vs. no) was additionally adjusted for in the models for propyl paraben; stimulation protocol (luteal phase vs. flare/antagonist) and the use of ICSI (yes vs. no) were additionally adjusted for in the models for methyl, propyl, and butyl paraben. [c]The p-trend value was calculated using the median of each quartile as a continuous variable. [d]No mediator met the inclusion criteria for any model, therefore we present a model adjusted for confounders only. [e]The LOD for butyl paraben was 0.2 ng/ml. [f]The p-value was calculated using above and below the LOD as categorical variables.
